# Management of Obese Patients with Cardiovascular Disease with Emerging Weight-Lowering Drugs: A Narrative Review

**DOI:** 10.3390/biomedicines14040778

**Published:** 2026-03-30

**Authors:** Alessandro Ciarloni, Gianmaria Salvio, Monia Bordoni, Gilberta Giacchetti, Giancarlo Balercia

**Affiliations:** Department of Clinical and Molecular Sciences, Polytechnic University of Marche, 60126 Ancona, Italy; g.salvio@staff.univpm.it (G.S.); m.bordoni@pm.univpm.it (M.B.); gilberta.giacchetti@ospedaliriuniti.marche.it (G.G.); g.balercia@staff.univpm.it (G.B.)

**Keywords:** weight loss, heart failure, overweight, new drugs, lifestyle

## Abstract

**Background/Objectives**: Obesity has a huge impact on global healthcare and economy. Consequently, the pharmaceutical industry has recently introduced novel anti-obesity drugs such as semaglutide and tirzepatide, which can yield remarkable weight reduction in patients, while also having significant cardiovascular benefits. **Methods**: Other weight-lowering medications are currently under investigation, and this narrative review provides an overview of the main novel drugs that are being tested. **Results:** These novel agents have different mechanisms of action, e.g., calorie intake reduction, increase in basal metabolism, and increase in muscle mass. **Conclusions:** In the future, obesity treatment is likely to become increasingly personalized, and further cardiovascular benefits could be expected. The combined use of different molecules could minimize their side effects, for instance, by minimizing muscle wasting observed during glucagon-like peptide 1 receptor agonists (GLP1-RAs) therapy. In our opinion, these highly effective drugs could represent a valuable addition to healthy lifestyle, as the evidence linking increases in muscle mass and basal metabolic rate to improved cardiovascular health is strongest when these changes are achieved through diet and regular physical activity.

## 1. Introduction

### 1.1. Global Impact of Obesity

Obesity is a chronic disease characterized by excess adiposity and defined as a body mass index (BMI) exceeding 30 kg/m^2^, according to the World Health Organization [1]. Its prevalence is continuously increasing and the impact on healthcare systems worldwide is becoming more relevant in recent years [2,3]. The social and economic costs of obesity have also increased due to associated comorbidities; indeed, the association between excess adiposity and diabetes mellitus type 2 (T2D), dyslipidemia, osteoarthritis, obstructive sleep apnoea syndrome, cardiovascular diseases and many other conditions is well known [4]. Excess body weight is also associated with premature mortality [5]. In 2021, obesity and overweight affected about 2 billion people worldwide [2]. Projections suggest that, by 2035, direct and indirect obesity-related costs could reduce global gross domestic product by 2.9%, equating to a $4 trillion loss [2,3].

### 1.2. Current Obesity Management

The Obesity Society recommends 5–10% weight loss in overweight or obese patients in order to obtain a significant reduction in cardiovascular risk factors such as blood pressure and glucolipid profile; however, further weight reduction leads to stronger cardiovascular benefits [6]. Although the first-line obesity treatment is food calorie restriction and physical activity, lifestyle interventions alone are often insufficient to obtain significant weight loss. Indeed, studies reported that only 40–65% of patients were able to obtain 5% weight loss at 6–12 months. Weight regains were then observed in more than 25% of patients after 2 years [7,8]. The main reason for failure is the inadequate adherence with long-term behavioural changes required to obtain slow weight loss [9]. To improve the success rate of lifestyle interventions, increasing attention is being directed to the development of pharmacological agents that are effective in reducing body weight. To this end, many new drugs with important therapeutic effects were commercialized in recent years. Since over 90% of T2D patients are affected by overweight or obesity, many drugs were first designed for T2D treatment and then adapted to obesity management considering the observed weight-lowering effects [10,11]. Glucagon-like peptide 1 receptor agonists (GLP1-RAs) are extensively used in obesity management, with one of the main agents being semaglutide [12,13]. Tirzepatide, a dual agonist acting on GLP1 and glucose-dependent insulinotropic polypeptide (GIP), was introduced later in the clinical practice and its weight-lowering effects seemed to be even more impressive [14]. GLP1-RAs can lead to weight reduction through the stimulation of brain satiety centres and delayed gastric emptying, which reduce food intake [15]. The double agonistic action on both GLP1 and GIP receptors enhances the physiological mechanisms previously described, resulting in a more significant weight loss [16]. That treatment can lead up to 20–22.5% weight reduction [17], similarly to bariatric surgery [18]. A less significant effect on weight loss was observed with sodium-glucose co-transporter 2 (SGLT2) inhibitors [19]. In this case, an increased urinary glucose excretion leads to caloric deficit, thus resulting in weight loss [19]. Systematic reviews and meta-analysis confirmed positive effects on weight in obese patients with or without T2D with mean difference in comparison to placebo ranging from −1.09 to 2.99 kg [20]. However, many other therapies are expected to become available in the future, considering the number of clinical trials involving weight-lowering drugs [21]. Non-injectable formulations of high dose GLP1-RAs are also under development: oral semaglutide 3–14 mg is already available for T2D treatment [22], and clinical trials on oral semaglutide 50 mg for obesity treatment have shown promising results. In a Phase 3 trial in obese patients without T2D, it caused 17.4% weight loss at 68 weeks, while also improving several cardiometabolic risk factors [23].

### 1.3. Cardiovascular Impact of Obesity

Among obesity-related comorbidities, cardiovascular diseases play a major role [4]. Every 4 kg/m^2^ increase in BMI lead to a 26% increase in risk of coronary heart disease (CHD) [24], independently from other comorbidities such as T2D, dyslipidemia or hypertension, and it also increases mortality risk after CHD [25,26]. In addition, a nearly linear correlation between BMI and blood pressure (BP) was observed [27]. Obesity doubles the risk of heart failure (HF) and stroke [28,29], with a 5% increase in risk of HF for each 1 kg/m^2^ BMI increase in men [30].

### 1.4. Cardiorenal Impact of Weight-Lowering Drugs

The availability of new weight-lowering drugs acting not only on body weight but also on cardiovascular comorbidities has strengthened the role of these drugs in the management of obesity. The positive cardiovascular effects of GLP1-RAs and dual agonists GLP1-GIP are probably related to combined weight loss, lowered BP and improved glucometabolic profile. This improves systemic inflammation, oxidative stress, and insulin resistance, that are all associated with endothelial dysfunction and progression of atherosclerotic process, potentially resulting in major adverse cardiovascular events (MACEs) [31]. A recent consensus statement underlined the central role of semaglutide and tirzepatide in obese patients affected by cardiometabolic comorbidity such as CHD, HF, hypertension and metabolic syndrome [32]. Indeed, both drugs have strong evidence on the improvement of systolic blood pressure, diastolic blood pressure (DBP), LDL cholesterol (LDL-C) levels reduction and HDL cholesterol (HDL-C) increase [31]. The SELECT trial indeed showed a 20% reduction in MACE in subjects treated with semaglutide compared to placebo [33]. In this trial, significant reduction in systolic blood pressure (mean decrease 3.3 mmHg), waist circumference and high-sensitivity C-reactive protein (hs-CRP, −37.8%) were also reported; these changes were independent from weight loss, suggesting a disease-modifying effect not related to weight reduction [33]. Effects on MACE are under evaluation for tirzepatide in the SURMOUNT-MMO trial, with results expected in 2027 [34]. However, treatment with tirzepatide proved to be non-inferior to dulaglutide, a drug that is known to strongly reduce the incidence of cardiovascular events [35], considering a composite outcomes of cardiovascular death causes in patients with T2D and atherosclerotic cardiovascular disease [36]. It also seems to reduce mortality and worsening of HF in patients affected by obesity and HF with preserved ejection fraction (HFpEF) [37]. Tirzepatide was also associated with important improvements in waist circumference (mean reduction −18.4 cm at 72 weeks), SBP (mean reduction −10.2 mmHg), and reduction in hs-CRP levels and markers of organ damage such as troponin T and NT-proBNP [38,39,40]. Semaglutide and tirzepatide have also shown to have important renal effects, probably related to the pleiotropic effects on BP and systemic inflammation; nevertheless, more information on mechanisms of action will be unravelled from studies like the REMODEL trial [41]. In the FLOW trial, semaglutide in T2D patients with chronic kidney disease reduced renal outcomes in comparison to placebo with or without concomitant SGLT2 inhibitors treatment [42]. The SELECT trial underscored positive effects of semaglutide on renal function also in obese patients without T2D [43]. In a post hoc analysis of SURPASS trials tirzepatide showed greater reduction in renal function decline in comparison to placebo and most commercialized antidiabetic drugs [44]. Positive renal effects of tirzepatide were subsequently confirmed in overweight/obese patients with or without TD2 [45]. Effects of SGLT2 inhibitors on cardiorenal outcomes are also sustained by the literature [46]. In overweight and obese patients, they were associated with a 28% reduction in cardiovascular events compared to placebo [46]. These effects seem to be related to natriuresis, reduced inflammation, and improved cardiac function and blood pressure [47]. SGLT2i also reported nephroprotective effects by improving renal function and albuminuria [48,49]. Finally, positive effects on incidence of MACE in obese patients treated with Orlistat, a weight-lowering drug inhibiting lipase activity, should be mentioned. These results derive from a nation-wide propensity score-matched cohort study on 36,876 obese patients taking Orlistat [50]. Despite the importance of results and the large sample size, the retrospective nature of the study and the source of the data from electronic records should be considered as they could limit the assessment of potential confounding factors on cardiovascular outcomes.

In this narrative review, we aim at giving an overview on the main pharmacologic classes under evaluation for obesity treatment that will probably become of common use in clinical practice, also describing their biological actions. Data regarding specific molecules of each drug class are reported. Evidence-based and theoretical cardiovascular effects are described.

## 2. Materials and Methods

An extensive literature search was conducted using Scopus and PubMed databases up to 30 December 2025. Only articles written in English were included. No restrictions on publication date or study design were applied.

The search terms included “cardiovascular”, “blood pressure”, “heart failure” “action”, “mechanism”, “biological”, “amylin”, “GIP agonism”, “GIP antagonism”, “Non-peptide GLP1-RAs”, “glucagon agonism”, “activin receptor antagonism”, “survodutide”, “mazdutide”, “retatrutide”, “orforglipron”, “danulipron”, “cagrilintide”, “cagrisema”, “bimagrumab”, “obesity”, “AMG133”, “Maritide”, “increase metabolic accelerator”, and “HU-6”. Boolean operators (AND/OR) were applied to combine search terms. References from retrieved studies were searched for further relevant literature.

We adhered to the principles of the SANRA assessment scale (Scale for the Assessment of Non-Systematic Review Articles) [51] to strengthen consistency and transparency of the paper. It is a tool for the final assessment of the quality of narrative reviews considering six points: (1) justification of the article’s importance for the readership, (2) statement of concrete aims or formulation of questions, (3), description of the literature search, (4) referencing, (5) scientific reasoning, and (6) appropriate presentation of data. In doing so, we selected articles that clearly described their objectives and the pathophysiological rationale underlying the study’s hypothesis. Where the same concept was addressed by several studies, we selected those that were methodologically more accurate and had the most robust study design. When selecting the reviews, preference was given to the most recent ones and to those that presented their conclusions in the most scientifically sound terms.

## 3. Main Weight-Lowering Drug Classes Under Investigation

### 3.1. Dual GLP-1 and Glucagon Receptor Agonist

#### 3.1.1. Mechanism of Action and Physiological Background

An interesting association of biological mechanisms is the combination of GLP-RAs and glucagon receptor (GCGR) agonists. The effects of GLP-1R agonism are well known: weight loss by reduction in appetite, direct action on brain satiety centres and delayed gastric emptying. It also reduces blood sugar by stimulating insulin and reducing glucagon secretion [44]. Glucagon is known to increase energetic expenditure, but its mechanism of action is not fully understood [52]. In animal models, an effect of glucagon in browning of adipose tissue and in the enhancement of thermogenesis was observed [52]. Glucagon seems to also enhance skeletal muscle thermogenesis [53,54]. An interesting hypothesis involves glucagon stimulating action on sympathetic nervous system leading to increased energy expenditure [52]. Another important glucagon action is the promotion of lipolysis trough lipid oxidation [55]; it acts both on adipose and hepatic tissue, leading to positive effects on weight excess and liver steatosis [56]. In the end, glucagon is also involved in the satiation mechanism, thus strengthening the effect of GLP1 [57,58]. All of these positive effects of glucagon agonism lead to improved glucometabolic profile even considering the stimulus to glucose hepatic output deriving from glucagon agonism that is however counterbalanced by the GLP-1R agonism [59,60]. Survodutide and mazdutide, mammalian oxyntomodulin analogues, are examples of drugs with that interesting synergic action. In vivo preclinical studies found a simultaneous activation by survodutide of both GLP1 receptor and GCGR [61]. The potency of activation of GLP1 receptor is eight times higher than GCGR activation potency, allowing for the obtainment of an important weight loss without significant alterations in glycemic control [61]. GLP1-RAs action leads to weight loss by delaying gastric emptying and acting on central receptor in the hindbrain and hypothalamus related to hunger and satiety [62]. However, the dose-dependent body weight reduction observed during survodutide treatment is not only related to reduced food intake but also to increased energy expenditure and basal metabolic rate [63].

#### 3.1.2. Clinical Evidence

Both survodutide and mazdutide were associated with a significant reduction in body weight in comparison to placebo in clinical trials, up to 13.8% and 12.6% [64], respectively. Mazdutide reported improved cardiovascular risk factors reducing systolic and DBP, total cholesterol, LDL-C, glycated hemoglobin (HbA1C), and triglycerides in comparison to placebo [64]. Similar results on cardiovascular profile were obtained with survodutide; indeed, a systematic review and meta-analysis of six randomized controlled trials (RCTs) and 1272 patients found moderate reductions in total cholesterol, triglycerides, and BP in comparison to placebo. However, survodutide treatment was often discontinued mainly due to gastrointestinal adverse events [65]. Regarding metabolic dysfunction-associated steatohepatitis (MASH), liver fat content was reduced up to 30% by servodutide in comparison to placebo [66]. Despite improvements observed on cardiovascular risk factor, data regarding impact on major cardiovascular outcomes are not available and need to be analyzed in future RCTs.

### 3.2. Triple Agonists GLP1-GIP-Glucagon

#### 3.2.1. Mechanism of Action and Physiological Background

GIP is secreted by K-cells in the duodenum and proximal jejunum after food intake and it stimulates insulin secretion in combination with GLP1 [67]. GIP seems to have direct effects also on the central nervous system; indeed, GIPR was found in hunger and satiety-related hypothalamus regions [68]. Furthermore, GIP could stimulate lipid metabolism increasing insulin sensitivity of adipocytes [69]. GCGR reduces body weight not only by regulating hunger but also by increasing energy expenditure, as previously described [52,53]. Although these three hormones may represent reciprocal counter-regulatory mechanisms under physiological conditions, appropriately balanced triple pharmacological agonism can lead to significant synergistic effects [70]. Indeed, the triple agonist GLP1-GIP-glucagon retatrutide is expected to significantly impact the management of obesity and T2D, as already shown by first clinical evidence.

#### 3.2.2. Clinical Evidence

Indeed, in a Phase 2 trial on obese or overweight patients it led to 24.2% weight loss at 48 weeks [71]. It is the best result obtained with weight-lowering drugs to date. Retatrutide appears safe on cardiovascular profile with only a slight dose-dependent increase in heart rate peaking at 24 weeks and declining thereafter [71]. In this Phase 2 trial, exploratory cardiovascular endpoints were evaluated and improvements in systolic and diastolic BP, as well as in glucometabolic parameters (with the only exception on HDL-C), were reported. At 48 weeks, 72% of patients with prediabetes at baseline reported normoglycemic parameters. Discontinuation of at least one anti-hypertensive drug was also reported in 41% of patients treated with 8 mg of retatrutide [71]. The reduction in triglyceride and HDL-C levels seems to be dependent on GCGR agonism, reducing the concentration of angiopoietin-like protein 3/8 complex [72], a protein involved in the control of lipoprotein lipase activity [73]. Such results are also very promising on cardiovascular effects. However, future RCTs are needed to assess the impact on major cardiovascular outcomes, and first results are expected from ongoing Phase 3 trials.

### 3.3. Combined GLP1-RAs and GIPR Antagonists

#### 3.3.1. Mechanism of Action and Physiological Background

Interestingly, the association of GLP1-RAs and a GIPR antagonist seems to also reduce body weight. Indeed, the association of maridebart, a GIP antagonist, and cafraglutide, a GLP1-RA (named maritide, formerly AMG133) is under clinical experimentation for obesity treatment [74]. It is not clear how both agonism and antagonism of GIPR can lead to weight loss. One hypothesis is that prolonged agonism could lead to receptor desensitization with final antagonism-like effects. In this case, the weight loss observed with GIP agonism could be effectively related to receptor desensitization after chronic agonism acting as a functional antagonism, while the GIP antagonism could lead to direct weight loss [75].

#### 3.3.2. Clinical Evidence

In a Phase 2 trial (NCT05669599), weight losses of 17% and 20% were reached in obese patients respectively with or without T2D. At the end of the 52-week study, the weight loss plateau was not reached and longer trials were ongoing. Maritide was also associated with significant improvement on cardiometabolic parameters such as BP, triglycerides, and high-sensitivity C-reactive protein (hs-CRP) [76,77]. However, significant effects on major cardiovascular outcomes still need to be evaluated.

### 3.4. Non-Peptide GLP1 Agonists

#### 3.4.1. Mechanism of Action and Physiological Background

Non-peptide GLP1-RAs, such as orforglipron and danuglipron, are a class of weight-lowering drugs specifically designed to overcome some limitations deriving from the biological structure of currently commercialized GLP1-RAs [78,79]. These are small molecules that exert the same biological action of their peptide counterpart by mimicking the same signalling pathways [80]. The majority of GLP1-RAs are injectable formulations [81] and the only available oral formulation (oral Semaglutide) does not reach the same weight-lowering efficacy [82]. Moreover, as a peptide, it must be taken in fasting conditions at least 30 min before the meal and not in association with other medications [82]. A non-peptide small molecule does not have such limitations and could make the therapy easier to carry out, encouraging adherence to treatment. In fact, small molecules are defined by their low molecular weight and chemical simplicity allowing them to exert biological effects not being influenced by metabolic processes or interactions requiring more complicated chemical structure [83].

#### 3.4.2. Clinical Evidence

Orforglipron treatment reported important weight loss in obese patients with and without T2D, respectively up to 6.6% at 26 weeks and 14.7% at 36 weeks in Phase 2 trials [84,85]. Similarly to classic GLP1-RAs [86,87,88,89], non-peptide ones seem to improve cardiovascular risk factor [90], even if major cardiovascular outcomes have not been evaluated yet. Orforglipron improved HbA1C up to 2.1% in diabetic patients in Phase 2 trials [84,85]. It also had positive effects on β-cell function and insulin sensitivity, evaluated with many different parameters such as HOMA-B, insulin-like growth factor binding protein 2, adiponectin, proinsulin, and the proinsulin/insulin ratio [91]. Indeed, in a sub-analysis of previously described Phase 2 trials [84,85], orforglipron treatment in patients with T2D and/or overweight or obesity was associated with a significant reduction in BP, triglycerides, LDL-C, ApoB, ApoC3, and hsCRP [90]. Most of these effects seem partially independent from the administered dose [90]. It must be remarked that danuglipron was withdrawn from the market due to frequent gastrointestinal side effects leading to high discontinuation rates [92].

### 3.5. Amylin Analogues

#### 3.5.1. Mechanism of Action and Physiological Background

Amylin is a pancreatic peptide that is co-secreted with insulin after food intake [93]. It belongs to the calcitonin family and induces satiation through its action on calcitonin receptor-expressing neurons in the hindbrain and hypothalamus and delaying gastric emptying [94,95]. Preclinical evidence suggests that it could also reach other brain regions like the hypothalamus (arcuate nucleus and parasubthalamic nucleus) and midbrain (lateral dorsal tegmentum and ventral tegmental area) [96]. It also acts in glucose control after food intake by reducing glucagon secretion [94,95]. Amylin is thought to have a role in the cardiovascular system causing vasodilatation, and reducing blood pressure in rodents; this effect seems to be exerted by the interaction of GCGRs [94]. Amylin seems to also increase leptin sensitivity in obesity [97] and to increase energy expenditure in animal models [98,99]. Amylin analogues are a class of drugs aiming at mimicking human amylin action to reduce food intake [100].

#### 3.5.2. Clinical Evidence

The amylin analogue cagrilintide, at 4.5 mg dose, was associated with greater weight loss than liraglutide 3.0 mg (respectively 10.8% and 9.0%, (*p* = 0.03) after 26 weeks of treatment) [101]. It also reduced triglycerides and very-low-density lipoprotein cholesterol similarly to liraglutide and significantly more than placebo [101]. Mechanisms of action of amylin analogues and GLP1-RAs are similar and complementary. Indeed, the association of cagrilintide and semaglutide (named CagriSema), reached better outcomes in weight loss (up to 20.4–22.7% in comparison to baseline) and glucometabolic parameters than the single drugs [102]. Preclinical evidence suggests that this combination therapy not only decreases food intake but also increases energy expenditure [103]. In the REDEFINE 1 trial, cagrisema was associated with improved cardiovascular outcomes such as BP, HbA1C, and C-reactive protein in obese or overweighted patients without diabetes mellitus [104]. However, properly designed randomized clinical trials are needed to evaluate to impact of cagrilintide and CagriSema on major cardiovascular outcomes.

### 3.6. Activin Receptor Antagonists

#### 3.6.1. Mechanism of Action and Physiological Background

The main mechanism of action of activin receptor antagonists, such as bimagrumab, is the inhibition of muscle wasting, leading to lean body mass (LBM) hypertrophy. Activins and myostatin are TGF-β family ligands that negatively regulate skeletal muscle mass binding activin receptors. That way, they are able to inhibit muscle mass growth and differentiation [105]. Bimagrumab is a dual-specific antagonist anti activin receptor type IIA (ActRIIA) and type IIB (ActRIIB). The first signalling described was trough ActRIIB leading to muscle wasting and cachexia in mice. However, the maximum effect in muscle hypertrophy was observed when both ActRIIA and ActRIIB were blocked as reported in genetic evidence on both receptor genes deficient mice [105].

#### 3.6.2. Clinical Evidence

In 2021, in a Phase 2 randomized controlled trial (RCT) on patients with T2D and BMIs between 28 and 40, bimagrumab led to significant reduction in body fat mass [−20.5% vs. −0.5% (*p* < 0.001)] and increase in lean body mass (LBM) [3.6% vs. −0.8% (*p* < 0.001)] in comparison to placebo [106]. Notably, despite an expected positive action of bimagrumab on glycemic control, increased insulin sensitivity observed in mice in the short-term (40 h post-injection), was not confirmed in the long-term (21 days post-injection), indeed a disruption of glycemic control was observed probably due to increased glucose hepatic output [107]. However, in humans, the bimagrumab-related changes in body composition resulted in improved insulin sensitivity evaluated with hyperinsulinaemic–euglycaemic clamp and intravenous glucose tolerance test [108]. A 2024 systematic review and meta-analysis of seven RCTs confirmed the effectiveness of bimagrumab in increasing thigh muscle volume and fat-free body mass and decreasing body fat mass in comparison to placebo [109]. In addition, evidence regarding effects of bimagrumab on cardiovascular outcomes are still missing.

### 3.7. Controlled Metabolic Accelerator

#### 3.7.1. Mechanism of Action and Physiological Background

HU-6 is a controlled metabolic accelerator, a class of weight-lowering drugs that increases energy expenditure by influencing electron transport chain and promoting mitochondrial uncoupling [110].

#### 3.7.2. Clinical Evidence

HU-6 seems to improve obesity-related complications such as T2D, MASH, hypertriglyceridemia, and HFpEF [111]. In a Phase 2 trial on patients with HFpEF, a 3.1 kg (−1.3% from baseline) mean weight loss (preferentially fat mass) was observed in comparison to 0.2 kg of placebo group after 3 months. Nevertheless, fat-specific weight loss improvements in functional status were not observe in HFpEF obese patients [112]. Longer studies are needed to properly evaluate such outcomes. The more significant weight loss observed was −1.49% from baseline in patients with non-alcoholic fatty liver disease and high BMI [111]. Moreover, improvements in cardiometabolic parameters such as BP, lipidic profile and cardiac structure profile were observed [76,113].

**Figure 1:**
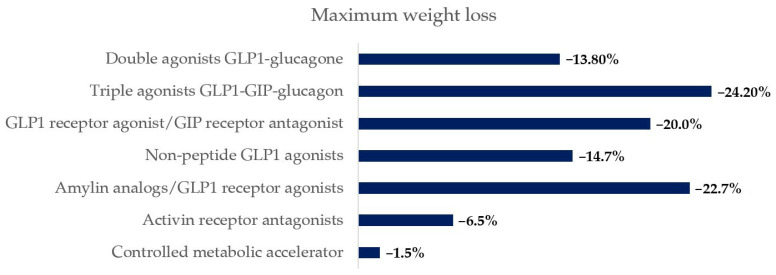
Maximum percentage weight loss reported for the main drug classes described [64,71,84,102,106,111,113,115].

**Figure 2:**
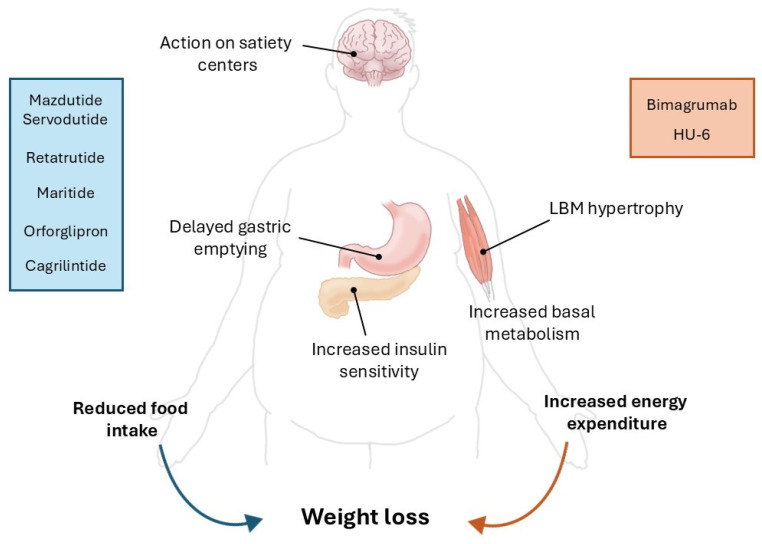
Different mechanisms of actions of anti-obesity drugs leading to weight loss. LBM = lean body mass.

Main mechanisms of action, maximum weight loss achieved and cardiovascular evidence of drugs classes previously described are reported in Table 1, Figure 1 and Figure 2.

## 4. Possible Synergic Effects of Different Drug Classes

Evidence on cardiovascular protective effects of many of the currently commercialized anti-obesity drugs are well supported by recent literature. Novel weight-lowering drugs have potential important cardiovascular effects that need to be investigated in properly designed clinical trials. The availability of drugs with different mechanisms of action provides fertile ground for investigating possible combinations of different active compounds that could act synergistically on weight loss, glycolipid metabolism, and cardiovascular health, enhancing their beneficial effects and counteracting their side effects. In this regard, bimagrumab could be able to limit muscle mass loss during treatment with GLP1-RAs. In fact, loss of LBM is common during such weight-lowering medical treatment [116,117], being associated with worse cardiovascular profile [118], and a concomitant adequate protein intake and physical activity has been underscored in the literature [119,120]. It is key to also consider that sarcopenia, characterized by loss of skeletal muscle mass and function, is often found in obese patients and it is defined as “sarcopenic obesity” [121]. Among the negative effects of sarcopenia we found increased risk of T2D, cognitive impairment, osteoporosis and mortality [122]. Sarcopenia is also quite common in patients affected by chronic HF, with an incidence that is 20% higher in comparison to healthy individuals [123]. In the pathogenesis of sarcopenia, reduced blood flow, low grade systemic inflammation and oxidative stress seem to play a major role [124], and it has been suggested that sarcopenia and chronic HF may negatively interact [124]. In this perspective, the antioxidant effect of GLP1-RAs and anti-sarcopenic action of bimagrumab could be combined and added to the weight-lowering effects of both drugs, potentially providing significant cardiovascular protective effect on patients affected by chronic HF. Accordingly, in murine models, semaglutide treatment alone led to loss of both fat and LBM, while combination therapy with semaglutide and bimagrumab enhanced weight loss and led to a 10% increase in LBM, also improving exercise performance and circulating markers of adipose inflammation [125]. Phase 2 trials exploring bimagrumab in combination with semaglutide (NCT05616013) or tirzepatide (NCT06643728) for obesity treatment in humans are ongoing [126,127].

A combination therapy with oral non-peptide GLP1-RAs, such as orforglipron or danulipron, and bimagrumab could represent an alternative option to avoid a double injective therapy. In fact, these drugs have shown promising results on cardiovascular profile [90] and their effect, differently from oral GLP1-RAs [128], is not influenced by other concomitant therapies and they do not need fasting condition for the assumption [82].

In our opinion, a promising treatment option is represented by the combination of energy expenditure deriving from glucagon agonists like retatrutide [114] or survodutide [54] and increased LBM [129] obtainable with bimagrumab [130]. Increased energy expenditure, when obtained with physical activity, is associated with improved metabolic markers like HDL-C, triglycerides and insulin sensitivity [131,132] and to reduced MACE [133,134]. However, it must be considered that evidence regarding positive cardiovascular effects of energy expenditure mainly derives from epidemiological studies and data on RCTs are missing. More so, data on pharmacological-induced increased energy expenditure effects on MACE are lacking. The only available ones are those regarding the positive impact on cardiovascular outcomes of GLP1-RAs [33], that also increase energy expenditure. However, their prevalent effect on reducing energy intake represents an important confounding factor in the interpretation of such results.

Interesting observations could be made regarding weight loss and increased energy expenditure in correlation to the well-known obesity paradox, which is that obese patients affected by HF and other chronic diseases show better prognosis in comparison to non-obese ones affected by the same diseases [31,135]. A possible explanation could be the better nutritional reserve of obese patients, particularly regarding lipids for myocardial substrate [31,136]. However, this hypothesis is highly controversial and the paradox could be related to HF onset at younger age in obese subjects in comparison to lean patients [135,136]. Moreover, BMI is not the ideal parameter to evaluate patients with CHF where excess fluid volume is present, because it does not take in consideration fat-free mass that strongly correlates with cardiorespiratory fitness (CRF) in such patients [136]. The obesity paradox underlines the necessity of evaluating survival outcomes in CHF patients treated with new weight-lowering drugs.

## 5. Side Effects Needing Further Studies

Despite the promising positive effects on weight loss and on cardiometabolic profile, some of the previously described treatments have shown adverse effects that need to be properly addressed in future studies. Bimagrumab, whose mechanism of action is of particular interest because it is able to increase LBM, reduced voluntary physical activity in recent preclinical evidence. Indeed, long-term treated mice reduced volitional running up to 250% [107]. A recent meta-analysis on patients with sarcopenia treated with bimagrumab showed that, despite the increase in muscle mass, the treatment was not associated with improved muscle strength and better physical performance at the six-minute walking test, whereas an improvement was observed only in patients with worst parameters at baseline [109]. Results from a controlled trial on patients affected by chronic obstructive pulmonary disease, another important chronic disease associated with reduced muscle mass, are in line with these data. Indeed, bimagrumab improved LBM without significant effects on functional outcomes [137]. These results are important considering that the main parameter able to estimate the positive cardiovascular impact of physical activity seems to be the CRF. It is a functional parameter referring to the ability of cardiovascular and respiratory systems to supply increased oxygen requests during prolonged physical activity [138]. Low CRF was independently correlated to cardiovascular diseases and all-cause mortality [139,140]. Indeed, positive cardiovascular effects of physical activity derives not only from anabolism of skeletal muscle but mainly from adaptation mechanisms, of which CRF represents the main example. Other important adaptations following physical activity are improvements in endothelial function, increased bioavailability of nitric oxide, enlargement of blood vessels diameters and increased vagal tone leading to lower heart rate [134]. Concerns were also raised on possible interaction of bimagrumab with hormonal function considering that ActRII ligands act also on gonads and adrenal and pituitary glands. However, the only statistically significant alteration found to date is a decrease in FSH only in women that was not clinically relevant and was reversible after discontinuation of bimagrumab. On the other hand, no alterations in adrenal and gonadal androgenic secretion were found [141].

For what concerns CagriSema, despite the important effects on weight loss, it needs to be pointed out that 72.5% of patients reported gastrointestinal adverse events with onlyfew patients tolerating the maximum dose [142]. More studies are surely needed to address that side effect, probably a more gradual titration could be useful to limit it. Similar problems with high discontinuation rates were also reported for double agonists GLP1/glucagone [65].

## 6. Conclusions

The range of available weight-lowering drugs is becoming wider, and it is expected to increase in the coming years. We did not focus on those drugs that are now far from clinical practice and/or with less physiologically predictable cardiovascular effects as cytokines (e.g., CIN-109), endocannabinoid receptor antagonist (e.g., monlunabant), centrally acting psychoactive agents (e.g., tesomet) and non-absorbed gastrointestinal agents (e.g., GLY-200) [29]. Drug therapies which use is currently reserved only for specific rare diseases, such as Melanocortin 4 receptor agonists, have been excluded from the review [29]. These drugs may act synergistically, limiting their side effects. Positive cardiovascular effects are also expected; nevertheless, results regarding major outcomes are currently lacking. In the context of highly effective personalized therapies, the real challenge for the future will be to maintain high awareness to the importance of diet and physical activity. This is important considering that the association between increased basal metabolic rate and improved cardiovascular profile mainly derives from increased physical activity. Furthermore, it is unclear whether pharmacologically induced muscle mass increase is associated with improved physical performance.

## Figures and Tables

**Table 1:** Mechanism of action and data on weight loss and cardiovascular outcomes of main weight-lowering drug classes described.

Drug Class	Mechanism of Action	Maximum Weight Loss	Cardiovascular Effects
Double agonists GLP1-glucagone	Delayed gastric emptying, action on hunger and satiety centres, increased energy expenditure and insulin sensitivity [62,63]	−13.8% [64]	Reduced systolic and diastolic blood pressure, total cholesterol, LDL cholesterol, glycated hemoglobin, and triglycerides in comparison to placebo [64,65]. No data on major cardiovascular outcomes
Triple agonists GLP1-GIP-glucagon	Delayed gastric emptying, action on hunger and satiety centres, increased energy expenditure and insulin sensitivity [52,68,114]	−24.2% [71]	Improved systolic and diastolic blood pressure as well as in glucometabolic parameters (with the only exception on HDL cholesterol) were reported [71]. No data on major cardiovascular outcomes
GLP1 receptor agonist/GIP receptor antagonist	Not fully understood	−20% [115]	Improved cardiometabolic parameters such as blood pressure, triglycerides, and high-sensitivity C-reactive protein (hs-CRP) [115] No data on major cardiovascular outcomes
Non-peptide GLP1 agonists	Delayed gastric emptying, action on hunger and satiety centres, increased insulin sensitivity [80]	−14.7% [84]	Reduction in blood pressure, triglycerides, low-density lipoprotein (LDL) cholesterol, glycated hemoglobin, insulin resistance, ApoB, ApoC3, and high-sensitivity C-reactive protein [90,91] No data on major cardiovascular outcomes
Amylin analogues/GLP1 receptor agonists	Delayed gastric emptying, action on hunger and satiety centres, increased energy expenditure and leptin and insulin sensitivity [94,95,97,99]	−22.7% [102]	Improved blood pressure, triglycerides, low-density lipoprotein (LDL) cholesterol, triglycerides, glycated hemoglobin, and high-sensitivity C-reactive protein [104] No data on major cardiovascular outcomes
Activin receptor antagonists	Inhibition of muscle wasting leading to lean body mass (LBM) hypertrophy and increased energy expenditure and insulin sensitivity [105,109]	−6.5% [106]	No direct data on cardiovascular outcomes
Controlled metabolic accelerator	Increased energy expenditure [110]	−1.5% [111,113]	Improved blood pressure, glucometabolic parameters and cardiac structure profile in patients with heart failure with preserved ejection fraction were observed [111,113]. No data on major cardiovascular outcomes

## Data Availability

No new data were created or analyzed in this study. Data sharing is not applicable to this article.

## Abbreviations

The following abbreviations are used in this manuscript:

BMI	Body Mass Index
GLP1-RAs	Glucagon-Like Peptide 1 Receptor Agonists
GIP	Glucose-Dependent Insulinotropic Polypeptide
T2D	Diabetes Mellitus Type 2
BP	Blood Pressure
CHD	Coronary Heart Disease
DBP	Diastolic Blood Pressure
LDL-C	LDL Cholesterol
HDL-C	HDL Cholesterol
MACE	Major Adverse Cardiovascular Events
HF	Heart Failure
HFpEF	Heart Failure with Preserved Ejection Fraction
SGLT2	Sodium-Glucose co-Trasporter 2
GCGR	Glucagon Receptor
RCTs	Randomizes Controlled Trials
MASH	Metabolic Dysfunction-Associated Steatohepatitis
GIPR	Glucose-Dependent Insulinotropic Polypeptide Receptor
hs-CRP	High-Sensitivity C-Reactive Protein
ApoB	Apolipoprotein B
ApoC3	Apolipoprotein C3
HbA1C	Glycosylate Hemoglobin
LBM	Lean Body Mass
ActRIIA	Activin Receptor Type IIA
ActRIIB	Activin Receptor Type IIB
TGFβ	Transforming growth factor beta
CRF	Cardiorespiratory Fitness
FSH	Follicular Stimulating Hormone
